# A MAC Protocol to Support Monitoring of Underwater Spaces †

**DOI:** 10.3390/s16070984

**Published:** 2016-06-27

**Authors:** Rodrigo Santos, Javier Orozco, Sergio F. Ochoa, Roc Meseguer, Gabriel Eggly, Marcelo F. Pistonesi

**Affiliations:** 1 Department Electrical and Computers, Universidad Nacional del Sur, CONICET, Bahia Blanca 8000, Argentina; jadorozco@gmail.com (J.O.); gmeggly@gmail.com (G.E.); 2 Computer Science Department, Universidad de Chile, Santiago 8370459, Chile; sochoa@dcc.uchile.cl; 3 Department of Computer Architecture, Universidad Politécnica de Catalunya, Barcelona 08034, Spain; meseguer@ac.upc.edu; 4 Department of Chemistry, Universidad Nacional del Sur, CIC, Bahia Blanca 8000, Argentina; mpistone@criba.edu.ar

**Keywords:** underwater sensor networks, underwater monitoring, MAC protocol, acoustic transmission

## Abstract

Underwater sensor networks are becoming an important field of research, because of their everyday increasing application scope. Examples of their application areas are environmental and pollution monitoring (mainly oil spills), oceanographic data collection, support for submarine geolocalization, ocean sampling and early tsunamis alert. The challenge of performing underwater communications is well known, provided that radio signals are useless in this medium, and a wired solution is too expensive. Therefore, the sensors in these networks transmit their information using acoustic signals that propagate well under water. This data transmission type not only brings an opportunity, but also several challenges to the implementation of these networks, e.g., in terms of energy consumption, data transmission and signal interference. In order to help advance the knowledge in the design and implementation of these networks for monitoring underwater spaces, this paper proposes a MAC protocol for acoustic communications between the nodes, based on a self-organized time division multiple access mechanism. The proposal was evaluated using simulations of a real monitoring scenario, and the obtained results are highly encouraging.

## 1. Introduction

The ocean covers 71 percent of the Earth’s surface and contains 97 percent of the planet’s water, yet more than 95 percent of the underwater world remains unexplored. UNESCO states that over 90 percent of the Earth’s habitable space is within the ocean, and near 80 percent of all life is under the ocean surface. There are quite dramatic projections stating that by the year 2100, more than half of the marine species may stand on the brink of extinction [1]. This shows only some of the several reasons that we have to deploy underwater technology that helps us study several phenomena that affect our lives and the health of our planet.

In this scenario, the underwater sensor networks could provide valuable information that helps us address some of these challenges [2]. However, underwater radio communications are practically unfeasible, especially in the ocean, because radio signals suffer a strong attenuation, limiting the effective communication to a few meters. Acoustic signals instead are capable of traveling for long distances, depending on the power used for the transmission and the physical characteristics of the medium. Therefore, the communication feasibility depends on several variables, such as the carrier communication frequency, the point-to-point distance, the chemical composition of the water, the topology of the seafloor, the temperature of the water, the depth of sensor placement and the spreading pattern [3].

Underwater acoustic sensor networks (UWASN) have several challenges that should be considered for a successful implementation. The first one is related to the lossy nature of the channel. Although acoustic signals can propagate well beneath the water, they suffer an important attenuation due to the absorption; and the signal amplitude attenuation is proportional to the distance and the frequency of the signal. There are some other factors contributing to the attenuation, like the scattering and reverberation in the surface, because wind moves the reflection point in the surface. There is also reflection in vessels that may introduce a Doppler effect in the signal. Moreover, the geometric spreading of the signal produces path loss in the transmission; regardless, we are using spherical (that is common in deep water) and cylindrical spreading (that is more common in shallow water) [4].

The second challenge to address refers to the nature of the acoustic signal used for transmitting the information. There are several man-made noises present in the ocean and especially in the surf zone, where it is usual to have a higher density, vessel traffic, sport activities and city noise. Another source of noise is provided by the environment itself; i.e., waves, winds, rains, animals and even seismic noise that may interfere with data transmission in the acoustic sensor network.

The third challenge is related to the multi-path propagation of the signals. Different signal paths may interfere between each other by means of the inter-symbol interference. The vertical links usually have little dispersion, so it is not common to have multi-path interference; however, in horizontal channels, the spread may be significant, and therefore, different signal paths may interfere among them.

The fourth challenge to be addressed is the energy consumption. Underwater sensor networks are battery powered, which also represents a major concern during its operation. The energy consumption during standby, data sensing and data reception is low in commercial modems, but in data transmission, the consumption depends on the distance that the signal should travel. The relationship between transmission power versus distance follows a quadratic function. One way to save the energy in these networks is by transmitting at higher baud rates for short periods of time. On the other hand, transmissions through long distances require high power and lower baud rates; thus, high data volumes result in being incompatible with large distances under the power consumption point of view. Considering that in sensor networks, the data volume is usually low, and assuming average ocean parameters, the speed of sound in the water is close to 1.5 km/s; the carrier frequency is between 20 kHz and 70 kHz for distances larger than 1 km. The operating range to maintain sustainable communications at a reasonable baud rate, and with low corrupted data rates, is limited to 2 km point-to-point. The variance in the transmission times introduces uncertainty; therefore, the round trip time for a message is usually pessimistically evaluated, degrading the performance of the system [3]. Usually, underwater modems are built around a piezoelectric device, and the physical layer modulation scheme is based on frequency-shift keying with frequency-hopping (FH-FSK) [5].

There is a transversal component in these sensor networks that can be used to help address most of these challenges. This component is the medium access control (MAC) protocol used by the UWASN. Considering the needs and the challenges to implement underwater acoustic sensor networks and the limitations that the current approaches for implementing a MAC protocol for this type of network, this paper proposes an extended medium access control protocol based on time division multiple access (TDMA), but specifically for UWASN. The paper presents Underwater Self-organizing TDMA (UWSO-TDMA), a new hierarchical synchronization procedure that is performed following a top-down approach. The frame length and slot allocation process is made in a distributed way. Within each hop, only one node acts as a synchronization node and works as a sink of a particular sub-frame. Once the slot selection procedure is finished, the frame is reversed, so in just one frame, the messages in the leafs of the tree may reach the root or sink. The proposed approach prevents the hidden station problems. To the best of the authors knowledge, similar proposals have not been previously reported in the literature.

The rest of the paper is organized in the following way. In Section 2, a short review of previous work is presented. In Section 3, the proposed MAC protocol is presented. In Section 4, a theoretical application example is presented, and in Section 5, a real application scenario is described. In Section 6, the evaluation process of this proposal is presented and discussed. Finally, in Section 7, we present the conclusions and future work.

## 2. Related Work

Underwater acoustic sensor networks (UWASN) has several aspects to consider, which are not yet defined, like the physical layer selection, link layer protocols and network and transport layer protocols [6,7,8,9]. The literature shows three main approaches to implement the medium access control protocol (MAC): frequency division multiple-access (FDMA), carrier sense multiple access with collision avoidance (CSMA/CA) and time division multiple access (TDMA) [10]. The first one is not suitable for underwater acoustic transmission, due to the narrow bandwidth in underwater acoustic channels and the vulnerability of limited band systems to fading and multi-path. CSMA/CA has been proposed in a previous work [11,12,13], but it is limited by the hidden and exposed station problem and the high propagation and variable delay. Long time periods are needed in each message transmission to guarantee that there have been no collisions degrading the overall performance. Finally, the TDMA schemes have problems with the synchronization, delay and unused bandwidth. However, for real-time transmissions, it is the only option that can provide bounded delays, although it is necessary to leave several slots empty. In this section, we discuss previous work mainly related to the medium access mechanism and its appropriateness for being used in underwater acoustic sensor networks.

In [14], the authors proposed multiple access collision avoidance (MACA), a protocol based on the use of short messages request to send (RTS) and clear to send (CTS), followed by the sequence of messages DATA and ACK. This mechanism has been successfully used in the Seaweb project [15]. Before sending data, the node should reserve the channel by issuing an RTS, and after that, it has to wait for the CTS answer. Nearby stations also listen to the request and wait for the answer. In the case that a neighbor station does not listen to the CTS answer, it means there is no interference in the receiver and that the transmission may proceed solving in this way the exposed problem. Any station, other than the original RTS sender, on hearing CTS will defer its transmission. In the case of collisions of RTS messages, the nodes use a binary backoff algorithm to solve the conflict.

In [16], the authors extend the MACA protocol and named it MACA for wireless (MACAW). They propose a less aggressive backoff algorithm for the link layer introducing the sequence RTS-CTS-DATA-ACK. In lightly-loaded scenarios, this mechanism has less performance, but with high loads, it has much better throughput and fairer allocation.

In [17], another MACA-based protocol is proposed. In this case, the floor acquisition multiple access protocol requires that every transmitting station should acquire the floor control (in a wireless channel), before sending any data packet. Both the sender and the receiver should perform the collision avoidance to guarantee the control of the channel.

In [18], the authors introduce the UW-MAC for acoustic underwater sensor networks. The MAC scheme is based on CDMA with a novel closed-loop distributed algorithm to set the optimal transmit power and code length to minimize the near-far effect. The algorithm compensates the multi-path effect by exploiting the time diversity in the underwater channel, thus achieving high channel reuse and a low number of packet retransmissions. The protocol works both in deep and shallow water and involving static or mobile nodes.

Self-organizing TDMA (SO-TDMA) [19] and ad-hoc self-organizing TDMA (ASO-TDMA) [10] use a self-organized TDMA scheme for the access control. However, these protocols were conceived for VHF radio frequencies, and they are used for the localization of vessels and ships in the ocean.

Finally, TDMA-based MAC underwater acoustic network protocols strongly need time synchronization to properly operate. The accuracy of a stations’ clocks are affected by several inner and external factors, like changes in temperature and power supply voltage, shocks and aging, which are all present in an underwater networks. The accuracy of the clock crystal is given in parts per million (ppm). Typical accuracies found in simulations for time synchronization methods are 40 ppm. In [20], time synchronization protocols are analyzed.

The UWSO-TDMA proposed in this paper is a simple mechanism that uses TDMA. In contrast to the other MAC protocols, this approach can be used for real-time communications, as it is possible to bound the transmission delay. The distributed MAC protocol works with static nodes in a multi-hop network.

## 3. Description of the UWSO-TDMA Protocol

Sensor networks like the one proposed in this paper are oriented to the data gathering. This implies that while there are several possible sources of data (sensor nodes), all of the data are transmitted towards a few sinks. This approach simplifies the data propagation, as it is not necessary to provide a path between all of the nodes in the network. In Section 3.1, the model used for the network is presented. By now, it is enough to know that as data captured by end nodes are transmitted towards the sink, intermediate nodes aggregate their own messages and also those provided by other nodes.

The proposed protocol is based on the predictability of TDMA, rather than on the probabilistic CSMA/CA approach. The nodes use a particular slot within a frame, which is allocated in a partially distributed way, as will be later explained. This is a decentralized scheme where nodes are responsible for sharing the communication channel and the synchronization. In the case of the UWASN, the universal time coordinated (UTC) information is not available under the water; therefore, the synchronization source is usually a sonobuoy (SB) anchored in the region that is in charge of synchronizing the clocks of the underwater nodes, and it is used as a sink of sensed data. Frames repeat periodically. We assume that nodes generate information at a fixed rate $T_{f}$, which is the frame period, and all messages have the same duration. Although one of the underwater nodes may be used instead of the SB as the synchronization phase, this node will not have access to the UTC, and we intend to have the network synchronized with the rest of the world.

Since acoustic signals are attenuated, not all of the nodes listen to the SB synchronization slot. When a node is not within the SB transmission range, it is synchronized in successive phases by nodes already synchronized. To do this, the frame is divided into *m* sub-frames (SF). Within the first sub-frame, $SF_{0}$, only the SB transmits. In the next sub-frame, $SF_{1}$, the nodes listening to the SB can transmit and are used to synchronize nodes at two hops. In $SF_{2}$, the nodes at two hops of the SB can transmit. With this hierarchical distribution, nodes in $SF_{j}$ are synchronized by nodes in $SF_{j-1}$. Each sub-frame has only one synchronizing node that is selected following the procedure described in Section 3.2.1. As nodes at a two-hop distance do not interfere, they may share the same time slot in the sub-frame, and different sub-frames originating in different synchronizing nodes may transmit simultaneously. The length of the sub-frames depends on the number of nodes being synchronized. As the SB is not aware of the network deployment, the frame length is not defined until all of the nodes of the network have been synchronized and are allocated to a particular sub-frame. Sub-frame $SF_{0}$ has only one slot.

In this paper, the use of only one SB is discussed. Although it is possible to think of a network with more than one SB, which means several roots in the tree, there is not a direct benefit in doing this, and the complexity of the system is incremented. The analysis is beyond the scope of this paper.

### 3.1. Network Model

The network can be modeled as a directed graph G=(V,E), in which *V* is the set of nodes in the network and *E* the set of edges. If two nodes *u* and *v* are within transmission range, there is an edge connecting them, e=(u,v). The slot assignment problem is an extension of the graph coloring problem [21], where the goal is to color each node of the graph with the minimum number of colors, such that no adjacent nodes and two-hops neighbors have the same color. The authors avoid the use of sub-frames by considering a slot allocation based on a previously-computed template that considers the two hop distance. However, this approach does not consider the transmission delay in the assignment that makes the problem more complex, as there may be collisions even if nodes used different slots. The problem is similar to the L(2,1) labeling on graphs and the frequency assignment [22].

Allocating nodes to slots in a frame is a classical optimization problem that may be solved by integer linear programming or some custom heuristics. However, this approach assumes a complete knowledge of the network, a centralized allocation procedure that is difficult to adapt to changes in the nodes. The latency in the frame is minimum, as the frame length is also minimum. In this work, we proposed the use of a partially-distributed slot assignment that is based on local information and is performed in a hierarchical way by each synchronizing node, avoiding the problems that come from the transmission delay in acoustic signals. We introduce the use of sub-frames for the different layers for simplifying the allocation protocol. However, these sub-frames increment the latency of TDMA as the frame becomes larger in comparison with the optimal one. However, we prefer this structure, as the slot allocation is made in a distributed way, and the transmission order is reshuffled, periodically updating the active nodes list.

During the initialization phase, in which the nodes synchronize their clocks and follow the slot selection process, the graph representing the network topology is built. Moreover, each node is aware of its neighbors, the path to the next hop and, eventually, of the alternative paths for the case of link or node failures.

### 3.2. Initialization Phase

#### 3.2.1. Clock Synchronization

The clocks synchronization is of major importance in a TDMA MAC protocol [20,23]. Although the nodes count with a real-time clock (RTC), these usually have drifts; therefore, a synchronization process is necessary. Underwater acoustic signals propagate at an average of 1.5 km/s. With this speed, nodes within transmission range (but distant enough) receive the signals at significantly different times. Moreover, usually the time slot is shorter than the propagation delay; thus, the same message may be listened to by one node at a certain instant and later by another one. Table 1 presents the propagation delay for different distances. If the transmission is set at 9600 b/s, which is a standard transmission rate for acoustic underwater transmissions, the time required for transmitting 256 bytes is 0.213 s. Therefore, nodes at more than 500 m receive the message after it has been completely sent at the source node, and a collision may take place without being noticed by the involved nodes.

The synchronization protocol is hierarchical. First, the nodes within the transmission range of the SB are synchronized. Once all of them are synchronized, they begin synchronizing nodes within their transmission range, and so on. In what follows, a detailed explanation is provided for the set of nodes in direct contact with the SB. The process is repeated once the synchronization nodes for the different sub-frames are selected.

In the case of underwater acoustic signals, the transmission range is not easy to determine, as it depends on the power of the transmitter, the sensibility of the receiver, the carrier frequency, the signal to noise ratio and several other factors, like water temperature, salinity, the depth of the sensors and currents. In fact, a node may discover nodes in its transmission range, but not the actual size of it. However, for the purpose of advancing in the analysis, we consider that in the design stage, it is possible to compute the communication range based on standard parameters of the zone in which the sensor network will operate.

At startup time (initialization phase), the network uses a carrier sense multiple access (CSMA) protocol for the clock synchronization and slot selection. As more than one node may try to use the channel simultaneously, there may be collisions. Nodes are not aware of the presence of their neighbors and the SB (*synchronization node*); thus, they do not know when the synchronization message will arrive or the amount of answers it will receive. The SB (*synchronization node*) sends a *synch* message. This has an identification field (ID) for the node and the time stamp obtained from the UTC system. Nodes within transmission range of the SB (*synchronization node*) would receive the *synch* message at different instants depending on the delay, which is basically a function of the distance to the SB (*synchronization node*), as shown in Table 1. After the node receives the *synch* message, it responds with an *asynch* message. This message has an ID field for the node, the time at which the *synch* message was received and the time at which the *asynch* message was finally transmitted. Before transmitting, the node will sense the channel to detect activity; if it listens to some other node answering, it will wait and run a back-off algorithm. As the propagation delay is important, overlapped transmissions may be detected by the transmitting nodes or by the SB (*synchronization node*). If the node finds the channel free and transmits, the message may suffer a collision later without being noticed by the node. For this reason, all messages should be acknowledged by the destination node, in this case the SB (*synchronization node*). If no acknowledgment is received, the node assumes its message has been corrupted for some reason and the most probable one is a collision. Then, a back-off algorithm is used for retransmitting the message.

The back-off algorithm is similar to the basic ones used in CSMA networks. After the first collision is detected, the nodes involved choose successively and randomly a delay that varies from zero to 16 slots. In the first round, they choose between zero and one, in the second between zero and three slots. In the third round, they choose a delay between zero and seven slots and, finally, between zero and 15. These numbers may change if the nodes’ density in a particular area is high.

During the synchronization phase, the SB (*synchronization node*) is not aware of the amount of nodes listening and the transmission delay to reach them. It is for this that the SB (*synchronization node*) has to wait for a period of time large enough to allow the transmission of the nodes, as they may be waiting for the channel to be free. Given the maximum transmission range, the SB (*synchronization node*) waits for a time equivalent to 2(c+2) maximum propagation delays, where *c* is the expected number of nodes in direct contact with the SB (*synchronization node*). The duration of this period is a parameter that can be enlarged or reduced according to the previous knowledge of the network topology. For example, assuming a maximum transmission range of 500 m and three nodes in direct contact, the waiting time will be 3.33 s according to Table 1. After this period, the SB (*synchronization node*) sends a new *synch* message. This second message has the ID, time stamp and the IDs of successfully synchronized nodes and the adjusted time for each one. A node that has answered to the first *synch* message, but is not acknowledge in the second with the adjusted time, is not synchronized and should send again the *asynch* message.

**Figure 1:**
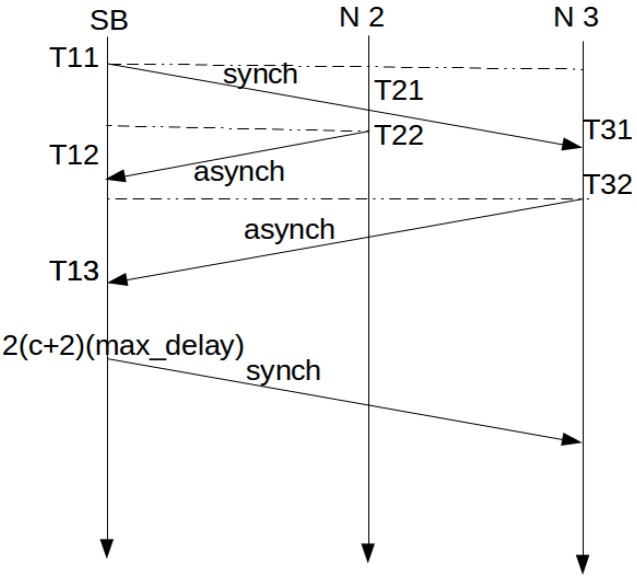
Synchronization protocol.

Figure 1 shows the message exchange and time evolution for the process when no collisions are produced.

In Figure 1, when Node 2 receives the *synch* message from the SB, it time stamps its own time as $T_{21}$. After that, in $T_{22}$, it is able to send the *asynch* message to the SB. Node 3 is further away and repeats the actions of Node 2. At $T_{12}$, the SB receives the *asynch* message from Node 2 and time stamps its own time. The *asynch* message contains $T_{11}$, $T_{21}$ and $T_{22}$. With this information, the synchronization can be performed at the SB.

$$\Delta t_{1}^{1}=T_{21}\;-\;T_{11}$$

$$\Delta t_{1}^{2}=T_{12}\;-\;T_{22}$$

The required correction is then obtained from:

(1) $$\Delta =\frac{\Delta t_{1}^{1}\;-\;\Delta t_{1}^{2}}{2}$$

This protocol is similar to the one proposed for time synchronization in the Internet, Network Time Protocol (NTP, RFC 5905) [24].

The described process follows a top-down approach from the SB towards the end nodes. If a node has two or more parents, it has an equal number of possible synchronization sources. In this case, the node with the lower transmission delay is chosen as the synchronization node. In the case that two or more nodes have equal delay, the node listened first is selected.

**Figure 2:**
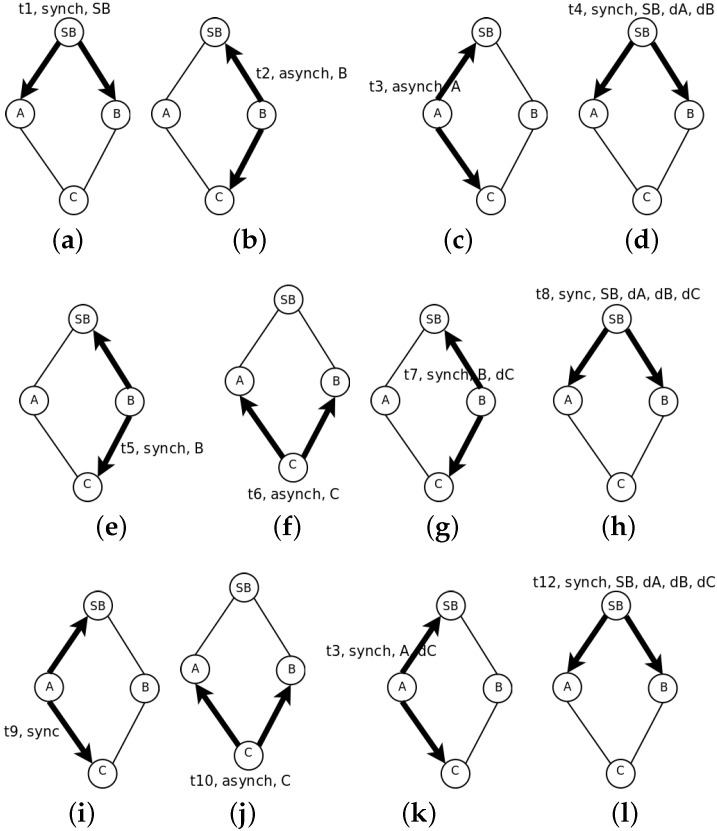
Example of the synchronization process: (**a**–**l**).

Figure 2 shows the synchronization process. First, the SB sends a *synch* message (Figure 2a). Both Nodes A and B listen to it. They are not aware of the presence of each other; therefore, they answer with the *asynch* message following the protocol described before. Let us suppose there is no collision and that Nodes B and A answer one after the other (Figure 2b,c). The SB recognizes both with a new *synch* message indicating the time correction and the computed distance to the SB (Figure 2d). With this message, both Nodes A and B learn about the presence of each other. As they both know the delay to the SB and this is the parameter used to order the synchronization process, Node B sends first a *synch* message (Figure 2e), and Node C recognizes it with an *asynch* (Figure 2f). Then, Node B sends a new *synch* message with the time correction for Node C and its delay to Node B (Figure 2g). The SB receives this message too and sends a new *synch* message acknowledging the presence of C (Figure 2h). Node A learns about Node C first by overhearing the exchange with Node B and after that, by the *synch* message from the SB. Now, it is time for Node A to send a *synch* message (Figure 2i). Node C listens to it and detects that A has less delay than B; therefore, it changes its synchronization node and answers with an *asynch* message (Figure 2j). This message is received by Node B, which learns the change of C from B to A. This latter node recognizes it with a new *synch* message indicating the time correction and its delay to A (Figure 2k). This message is also received by the SB that updates its tree structure. It sends the last *synch* message in which it indicates the time correction for each node and the delays to the SB (Figure 2l). After this, the slot selection process may proceed.

The synchronization process is used by overhearing nodes in the network to determine the set of neighbor nodes that may help during the slot selection process. Even if nodes are not within transmission range, they may discover their presence by the following *synch* messages. The synchronization process is similar to the leader election process [25]. For each sub-frame, only one node acts as the synchronization source. The set of synchronization nodes are saved in $S=\{s_{i}\}$. The set of nodes synchronized by $s_{i}$ is notated $V_{i}=\left\{v_{i1},v_{i2},\ldots ,v_{in}\right\}$; they are ordered by increasing transmission delay to $s_{i}$. If any $v_{ij}$ listens to more than one synchronization node, as shown in Figure 2, only one of them is selected, and the rest are saved as back up. In the *asynch* message, the node indicates with which node it is synchronized, and this information is passed to the upper layers up to the SB. The set of shared nodes that may be synchronized by more than one node is notated $Sh_{i}=\left\{v_{ij}\right\}$. The nodes with this condition indicate the presence of all possible synchronization sources to the upper layer. This information is used later in the slot selection process, as explained in Section 3.2.2. The information is confirmed in the following *synch* messages.

Once the synchronization nodes have been selected, the information flow from the SB to the leafs and from the leafs to the SB is fixed, and it will last until a new initialization process has begun.

#### 3.2.2. Slot Allocation

Once the nodes in the network are synchronized, the slot selection process can begin. This phase is again performed in a hierarchical way, beginning with the SB. The synchronization nodes are already selected for each sub-frame. The length of each sub-frame and the slot in which each node transmits should be defined. As the synchronization nodes already know which nodes are within their domain, they are able to setup the sub-frame length. With this information, they are able to allocate the slots in the following sub-frame. Messages sent by synchronization nodes are listened to both by downstream and upstream nodes; therefore, the network topology and sub-frames structure is propagated in both directions during the initialization phase.

**Figure 3:**
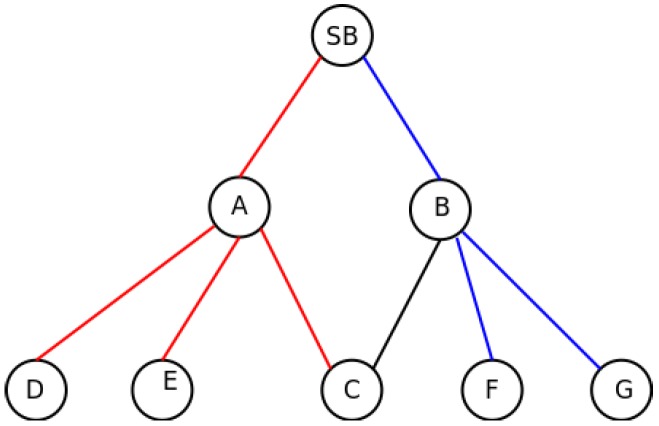
Slot allocation: Situation I.

We need to re-define the concept of simultaneous transmission. Two nodes transmit simultaneously if their messages produce a collision at some other node listening to both. To prevent this, the distance to the synchronizing node, or transmission delay already measured during the synchronization phase, should be used for ordering the slot allocation. The first slot is for the closer node and the last one for the one further away. In the case of Figure 2, Nodes B and A would use the first and second slots, respectively. Although this allocation order prevents the hidden station problem while transmitting messages from the nodes in sub-frame *i* towards the synchronizing node in sub-frame i−1, there may be collisions between nodes in the same sub-frame, but with different synchronizing nodes when a node (like C) uses more than one node for the synchronization. In Figure 3, the previous example is extended. In red, we mark a synchronization branch and in blue, the other. In this case, although Node C is synchronized by Node A and therefore the slot allocation is decided by Node A, the possible hidden station problem should be considered in Node B, as Nodes F or G may transmit simultaneously with C.

The described situation should be addressed by Nodes A and B. Both nodes know that C is within transmission range of each other, so they have to share the slot allocated to C in such a way that no collisions are produced. In these cases, the slot allocation criteria are changed. For each sub-frame level, a set of slots is reserved for those nodes that are connected with more than one synchronizing node (shared nodes). The amount of slots reserved for them is two times the number of nodes in that situation. The rest of the nodes are allocated after these reserved slots following the delay criteria. Within the reserved slots, each synchronizing node allocates the possible conflicting nodes. This allocation repeats the previous criteria. The first node in the previous sub-frame allocates first the possible conflicting nodes, leaving empty the rest of the reserved slots. The procedure is repeated for the rest of the synchronizing nodes. If a collision is detected after this allocation process, the nodes detecting it inform the previous layer of the situation, and the conflicting nodes are rearranged.

**Figure 4:**
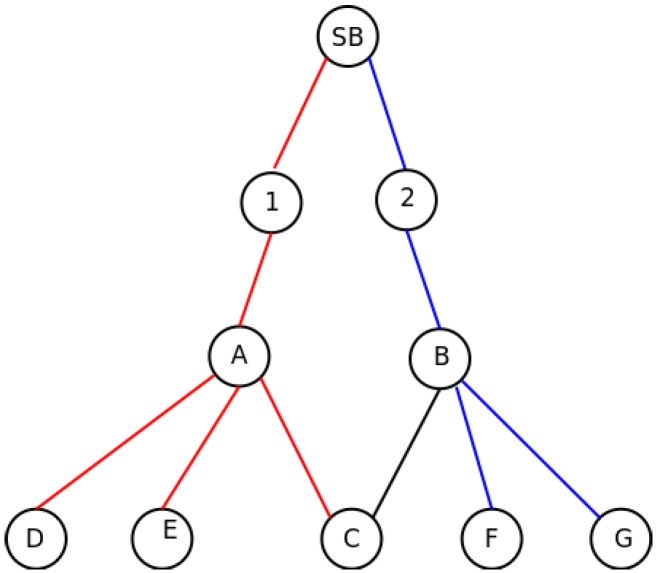
Slot allocation: Situation II.

In the case described in Figure 3, the synchronization nodes share the same sub-frame, as both of them are synchronized by the same node. In Figure 4, the situation is repeated, but in this case, both possible synchronization nodes are in different sub-frames, as they are not synchronized by the SB, but by Nodes 1 and 2, respectively. In this case, as explained in Section 3.2.1, Nodes A and B are aware of the presence of each other, because C informs about the presence of both of them in the *asynch* message. Since they are not under the same synchronization node, they know that they are in different sub-frames, as well. This information is passed to Nodes 1 and 2, so they should include as many empty slots as shared nodes exist in the subsequent sub-frames to avoid the hidden station problem. In the example, Node 1 will generate a two-slot sub-frame for Node A, and Node 2 will do the same with Node B. In this way, A and B should use different slots to prevent the collision in Node C.

After several iterations, the allocation should converge to a stable solution, as there are more available slots than nodes to use them. The number of free slots in the sub-frame helps with the initialization process, but it introduces more latency in the network for data transmission.

**Figure 5:**
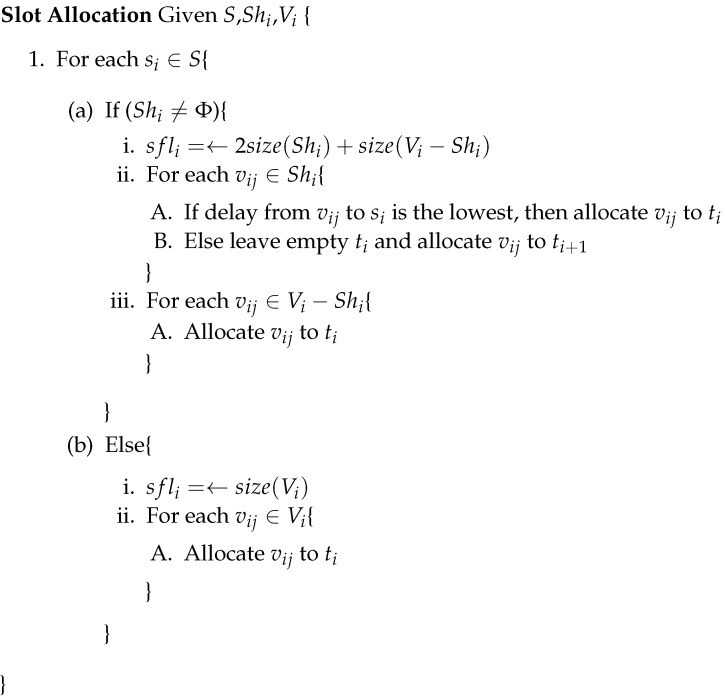
Algorithm for slot allocation.

A sub-frame synchronized by node $s_{i}\in S$ is notated $SF_{ji}=\left\{t_{1},t_{2},\ldots ,t_{sfl_{ij}}\right\}$, where subindex *j* indicates the hop number and *i* the synchronizing node, and $sfl_{ji}$ indicates the amount of slots within the sub-frame. Figure 5 presents the algorithm for the slot selection process. At this point, we can assure that the exposed station problem is not present in this configuration, because nodes within the transmission range of each other are allocated to different slots.

**Figure 6:**
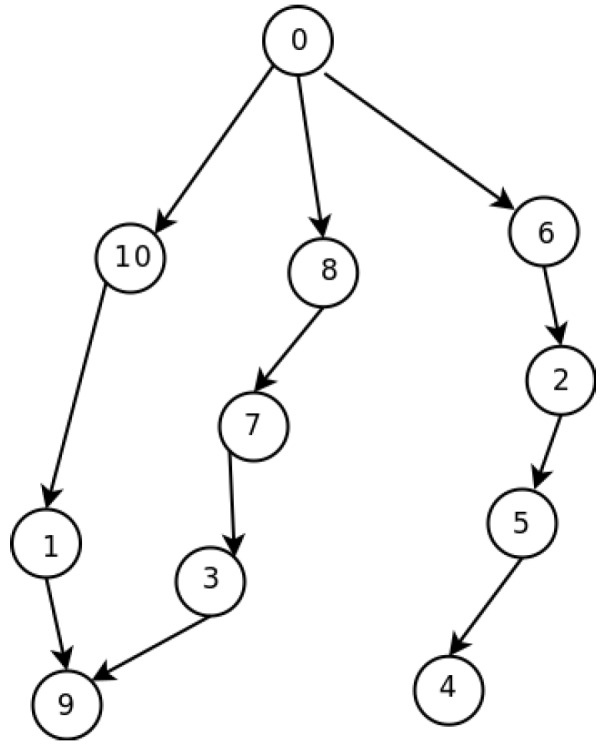
Network model example.

**Figure 7:**
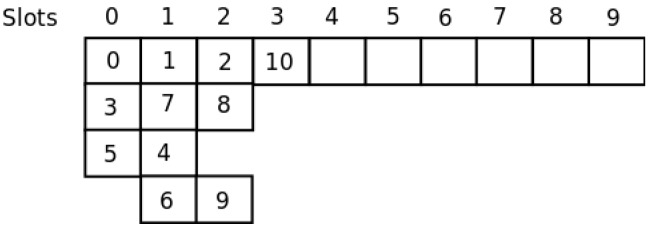
Frame.

**Figure 8:**
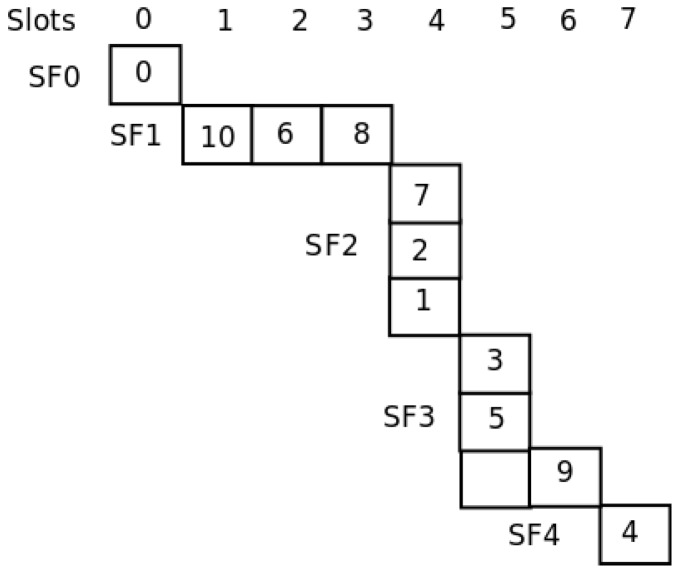
SO-UWSN frame.

For the network shown in Figure 6, in [21], the authors show the way in which their template allocates the nodes to slots in a global optimum way. Figure 7 shows the assignment. As can be seen, with only four slots, all nodes are able to send their messages to the sink. With our distributed method, the SO-UWSN allocates the slots according to Figure 8. It requires some more slots, incrementing the latency. However, the method is distributed, and no previous knowledge of the nodes’ deployment is necessary. As the protocol proposes also a periodic re-initialization of the network, dead nodes can be removed from the graph and eventually new ones incorporated.

### 3.3. Data Transmission Phase

**Figure 9:**
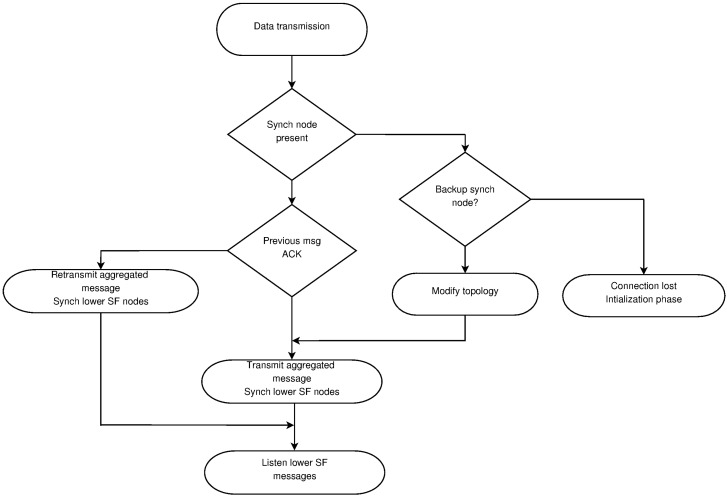
Data transmission flowchart.

Figure 9 shows the flowchart for the data transmission phase. In this phase, nodes transmit their data in the selected slot, together with the delay to the SB and depth. As all nodes within range overhear this information, they are able to keep an updated version of the network topology. As underwater transmission is prone to have data corruption, each message should be acknowledged by the synchronizing nodes in the previous sub-frames. If a message is not acknowledged, the sender retransmits it in the next frame. In the case a new message is generated, the information is aggregated or the old message is discarded, but this situation is flagged, so upstream nodes may notice the missing one. At the beginning of every frame, nodes wait for the synchronization message. If this message is not received with the acknowledgments of previous messages, the nodes in the sub-frame should assume that the synchronization node is out of work, and the backup synchronization node should be used if available. If there is not a backup, the node goes into the initialization phase.

Data messages differ from synchronization messages. The first ones only contain information about the process, while the second ones are used during the node synchronization and slot allocation processes.

### 3.4. Enhancements

The basic allocation policy contemplates only one slot per node. However, some nodes have more information to transmit, as they become the gateway for the previous sub-frame or upstream nodes. The synchronizing nodes aggregate the information from all of the nodes in the following sub-frame with their own information. In the case of a large tree structure, synchronizing nodes close to the SB will have a large amount of information to transmit and still use only one slot. In order to solve this situation, once the network topology and structure is learned by the clock synchronization procedure, some of the synchronizing nodes may be allocated more than one slot for transmitting the aggregated messages.

TDMA MAC protocols usually introduce an important delay, since messages require as many frames as hops to reach the sink. In cases like the one proposed here, it is useful to reverse the transmission order. As every node is synchronized and aware of the exact moment in which they should transmit, the frame may be turned around. In this way, a message starting in the farthest node to the SB in the network will be transmitted first, and as being received by the successive synchronization nodes, they are aggregated to the different sub-frame messages until reaching the SB in the period of just one frame. With this strategy, the delay introduced by the MAC is reduced to just one frame.

The previous enhancement facilitates another one, which is the possibility of implementing a streaming solution for this kind of network. In the actual state, the TDMA assumes there is a short transmission window in which all nodes are ready to receive and transmit followed by a long silent window in which all of the nodes sleep, providing good energy management. However, in certain cases or in particular scenarios, the reverse transmission order of the frame allows an almost continuous transmission of information. In fact, as the slot allocation guarantees that there are no collisions, a new frame may be started at two slots’ time distance. This may be used in the cases in which the slot time is not enough for sending the data collected at the nodes, for example audio or video streaming. Even more, the network may operate in different modes allowing low and high energy consumption according to some particular situation.

## 4. Application Example

**Figure 10:**
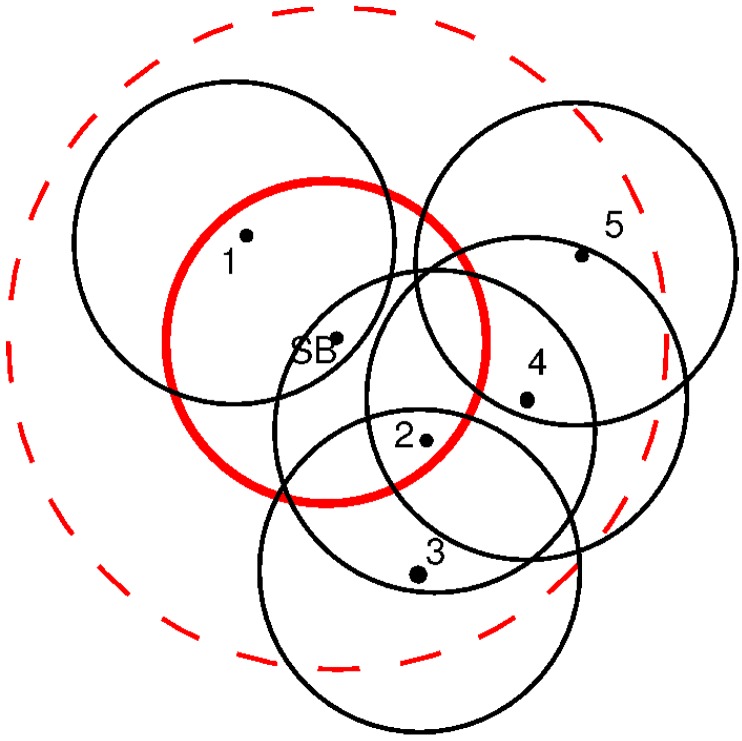
Case 1: deployment and transmission ranges.

Figure 10 presents an example with a possible distribution of nodes in a UWASN. The SB is the center of the red circle that marks the communication range of the SB, which we set to be 500 m. As can be seen, two nodes may be in direct contact with the SB, but not between them (e.g., Nodes 1 and 2). Both nodes synchronize with the SB. As they have almost the same delay to the SB, we assume Slots 1 and 2 are allocated to Nodes 1 and 2 in the $SF_{1}$, respectively. In the next hop area (dotted red circle), there are three more nodes. Like in the previous case, even if they have a similar delay to the SB, that is within the second hop, they have different synchronizing nodes and, thus, data paths. Node 2 synchronizes Nodes 3 and 4 in $SF_{2}$, and Node 4 synchronizes Node 5 in $SF_{3}$.

The slot allocation is trivial for this example. A message starting in Node 5 may need to go through Nodes 4 and 2 before reaching the SB. Instead, Node 3 is within range of Node 2, so messages beginning in Node 3 pass through Node 2 before reaching the SB. This example shows that even if Nodes 3, 4 and 5 are within a distance of two communication radios from the SB, the amount of hops needed to reach such a node is different for each one.

During this phase, nodes build the network topology to determine the path towards the SB. Each node in the system is allocated to a slot in the frame, and it informs about its depth, delay to the SB (if it is within one hop), the accumulated delay and the path towards the SB. In the example shown in Figure 10, Node 4 informs about the delay to the SB and the path through Node 2. In that way, Node 5 can determine its own delay to the SB and path.

**Figure 11:**
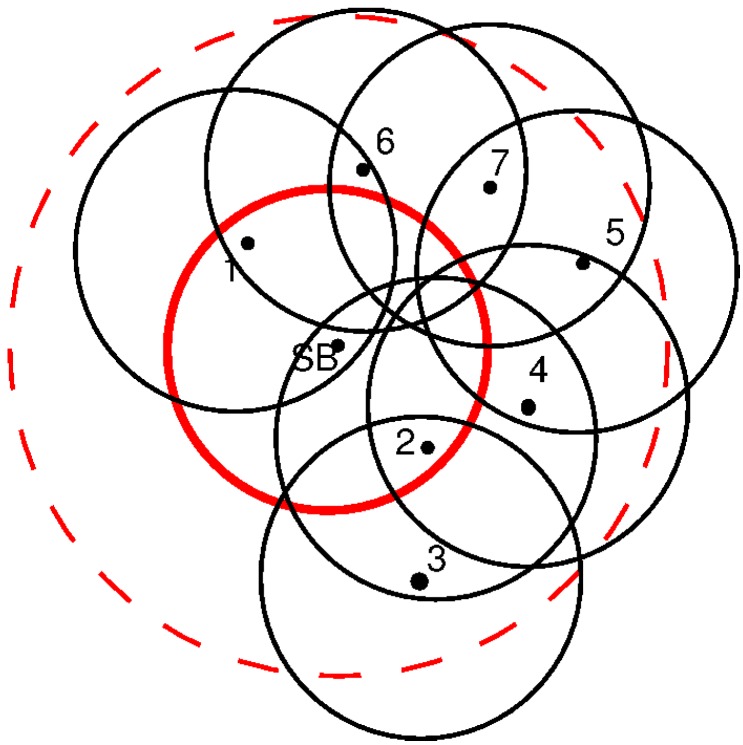
Case 2: deployment and transmission ranges.

In Figure 10, the Node 5 is the last node, and its messages have to go through Nodes 4 and 2 to reach the SB. In this case, Node 4 has to aggregate the information of Node 5 in its own message, and in the same way, Node 2 aggregates the information from Nodes 4 and 5 in its own message. Figure 11 extends the example incorporating new nodes. As the networks is re-initialized periodically, Node 1 will discover the presence of Node 6 and this last one the presence of Node 7 that is also discovered by Node 5. Let us assume that the delay between Nodes 6 and 7 is smaller than the delay between Nodes 5 and 7. Under these considerations, Node 1 synchronizes Node 6, and this last one synchronizes Node 7. In this case, the path from 6 to the SB is through Node 1. However, as can be seen, Node 7 listens to Nodes 5 and 6. Node 5 is in the third sub-frame (SF), but Node 6 is in the second one. The aggregation process is made only by nodes in the previous SF. When there is more than one node in the previous SF that listens to the messages from lower SF nodes, only one node aggregates and forwards the information to the next SF. The other nodes keep a copy of the information as a backup, in case there is a failure in the transmission.

**Figure 12:**
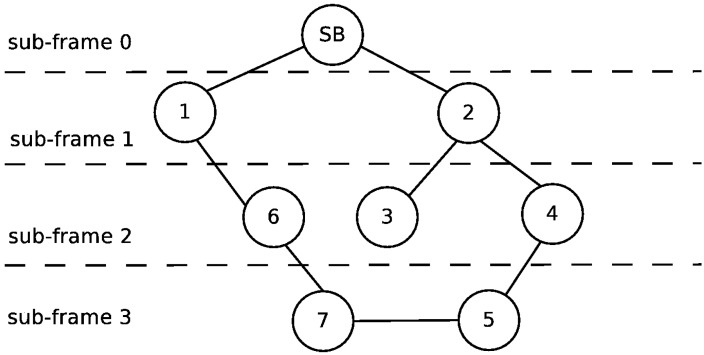
Topology of the network in Case 2.

**Figure 13:**
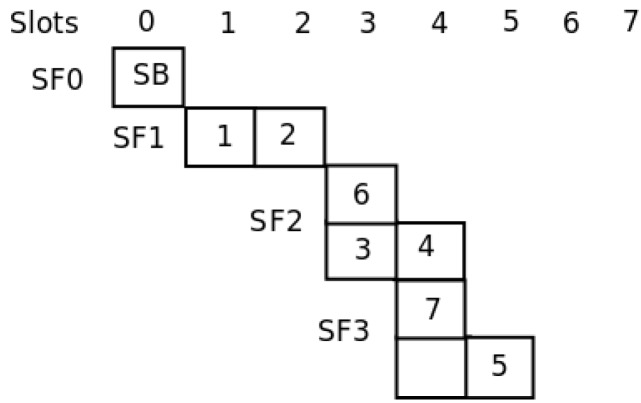
Slot allocation in the example frame.

Figure 12 presents the topology of the UWASN represented in Figure 11. The dotted lines represent the different sub-frames used in the transmission of messages. As can be seen, if Node 6 disappears, the network will be reconfigured, and Node 7 will be in a new sub-frame behind Node 5. In the same way, if Node 4 is turned off, Node 5 will send its messages through Node 7. Figure 13 illustrates the slot allocation in the example frame.

## 5. Application Scenario

UWSNs may be used in different application areas for monitoring, from military to environmental scenarios. As explained before, more than 70% of the Earth is covered by the ocean, and most of it is still unexplored. One of the hot topics in ocean research is related to the interaction of the sea and the coast in the presence of human activities. In this sense, we are using this proposal in the design of an underwater sensor network to monitor several variables in the Bahia Blanca (Argentina) estuary.

This city is the home of Ingeniero White port, which was originally used to export crops and fruits from a large area. In the last 30 years, this site has been surrounded by a large petrochemical nucleus, refineries and fertilizer plants, as well as deep water harbors. This means that periodical dredging, artisanal and commercial fisheries and oil and cereal cargo vessel traffic usually affect this area, as well as the direct impact of the city.

Several environmental studies have been done in the estuary, mainly in the inner part of it. For this reason, there is a large time-series database on physic-chemical parameters that has been assembled. These series are very important, as they provide information on the entry points of the main rivers and the main human activities through the city sewage. The estuary has a large mudflat with a rich ecosystem supporting a large population of wildlife, including migratory birds, crabs, fishes and mollusks. The increment of the port activity and the incorporation of new industrial activities have modified the ecosystem of the estuary.

**Figure 14:**
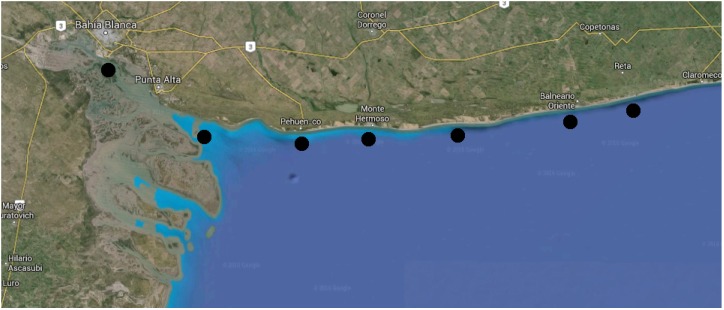
Satellite picture of the Bahia Blanca estuary.

At present, the influence of the port, the industrial activities and the city itself is being analyzed in the outer part of the estuary, especially in the surf zone outside the estuary. Several seaside towns at 50, 80 and 170 km away from the port are under analysis. For the first of these towns, Pehuen Co, it has been already proven that the ecosystem is under a severe effect of the port. The estuary has very strong tidal currents, and these affect the neighbor beaches with a large tidal amplitude. The other two, Monte Hermoso and Reta, are located further away, but most importantly, they are at open sea. Figure 14 shows a capture of the satellite image of part of the estuary with the city of Bahia Blanca and its port on the left and the seaside towns along the beach. The black circles represent the probable locations for the sensor networks.

The project tries to prove whether the port has modified the ecosystem of these beaches. For this, an underwater sensor network will be deployed in the area to measure different parameters, like water turbidity, spectral radiance, temperature, density, salinity, monitoring volatile organic and inorganic compounds. These parameters should be measured at different depths and distances from the coast and along several months to capture seasonal variations. Although it is possible to deploy a wired network, in the surf zone and under the effects of waves, it presents severe drawbacks. Besides, as these beaches have people living in the seaside towns, the sensors and cables may be broken.

Different clusters of nodes should be deployed along the coast to determine the presence of pollutants and variations in association with the tidal currents and the effect of wind. The first cluster should be deployed in the proximity of the port; a second group of sensors would be deployed in the mouth of the estuary, while the third one in Pehuen Co. Between Monte Hermoso and Pehuen Co, there are just 25 km with no river mouths in the middle. The fourth cluster will be anchored in Monte Hermoso. Two important river mouths are on the coast between Monte Hermoso and Reta, Sauce Grande and Quequén Salada. Intermediate clusters are deployed at these points to measure the influence of the rivers in the seaside. Finally, in Reta, there is also a small water stream mouth, the river El Gaucho.

**Figure 15:**
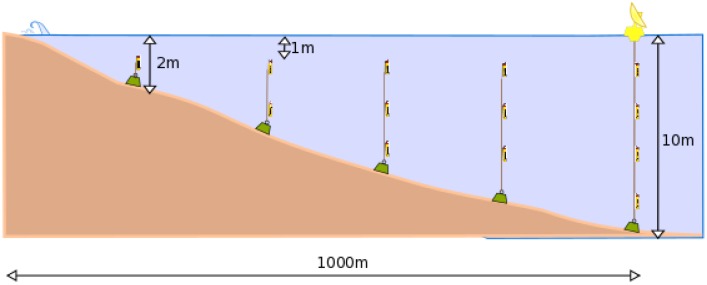
Network deployment at the seaside.

Each cluster is composed of several underwater nodes and one SB that acts as a gateway. Anchored at different depths, the nodes would cover an area of approximately 500 m starting in the low tide limit with a depth of 2 m and getting into the sea up to a depth of 10 m. The nodes will measure at 1 m, 2 m, 5 m and 10 m. The first cluster in the port area serves as a reference for the others. Figure 15 shows the approximate network deployment. Specifically, the sensors will measure: temperature, salinity, pH, turbidity, dissolved oxygen, dissolved nitrogen, dissolved nutrients (nitrate, nitrite, phosphate, silicate) and chlorophyll. All of these sensors may work on demand based on the flow injection analysis chemistry principles.

## 6. Evaluation Process

In this section, we evaluate the performance of the MAC proposal using the application scenario introduced in the previous section. We compare the general behavior of our proposal to the one of the MACA-U protocol [26]. For this evaluation process, we assume that each node has an acoustic range equal to 500 m; the carrier frequency is set to 40 kHz [27] and the transmission data rate to 9600 bits/s. Each slot takes 167 ms for 160 bytes. Figure 13 shows the frame structure after the initialization phase for the network topology represented in Figure 12. As can be seen, there are seven slots used. The complete frame is transmitted in 1.167 s.

Frames can start with different periodicity, for example every 10 s. In this case, the power demand (i.e., energy consumption) of the communication system in the node will be slightly more than 10%. After the initialization phase, a message from the farthest node (in our case, Node 7) requires three frames to reach the SB or 30 s. The ACK message is issued in three steps. First, Node 6 acknowledges the reception of the message to Node 7 and in the next frame; then, Node 1 acknowledges Node 6 in the following frame; and finally, the SB acknowledges the reception of the message from Node 1. If the frame reverse order is implemented, the message will reach the SB in just 10 s. In steady state, it is possible to send a message every 10 s, receiving the acknowledgment in the following 10 s, and the general utilization ratio of the network is close to 10%.

In the case of the MACA-U protocol, the nodes can transmit whenever they have information available, and they can lock the access to the acoustic channel, by issuing a RTS-CTS message with the next hop in the network. In this case, a timer is set with both, the time needed to send the message and the maximum delay for the message to reach the next node. Considering that the underwater acoustic signal propagates at 1.5 km/s, the time needed to reach a node in the limit of the transmission range is equal to 0.333 s; therefore, the maximum round trip $\tau_{\max}$ is equal to 0.667 s. The same time slot is kept for comparison purposes at 0.167 s. In case Node 7 has a message to send, it will send an RTS message and wait for $\tau_{\max}\;+\;t_{slot}\;=\;0.834$ s. If in this time, this node receives a CTS message, then it gains access and transmits its message. So, the total time needed by Node 7 to send its message to the SB is 3 s without collisions. However, Node 7 may be delayed by collisions with Nodes 5 and 6. In this case, and assuming only one collision with each one, the message from Node 7 will only be transmitted to Node 6 after 3 s. Node 6 can be delayed by collisions with Node 1 and 7. Therefore, considering again only one collision with each one, the message needs another 3 s to reach Node 1. In the last hop, Node 1 may have collisions with Nodes 6, 2 and the SB, and like in the previous cases, the message will be delayed for another 4 s. The end-to-end transmission time for a message originating in Node 7 is the sum of the partial delays, i.e., 10 s.

This simple example shows that the complexity of a collision avoidance algorithm for the transmission of information in networks with important delays may compromise its performance severely. Another important aspect is that the delay is not deterministic, showing great variations according to the network load. Instead, once the slot selection process is done, the TDMA approach guarantees a constant latency and transmission delay, making the message propagation predictable.

## 7. Conclusions and Future Work

In this paper, we have proposed the UWSO-TDMA MAC protocol for supporting underwater acoustic sensor networks. The mechanism is based on a TDMA protocol, but without including a master node or a fixed schedule for the nodes. In fact, each node selects the slot in which it will transmit in a distributed way. Time synchronization is kept through a sonobuoy that picks up the time from GPS or the Galileo system. Nodes that are not within the sonobuoy transmission range synchronize their clocks through retransmitting nodes. As the protocol is based on TDMA, transmission delay and latency are bounded, and the network can be used for transmitting messages with real-time constraints.

The proposal was evaluated using simulations that consider the monitoring of the Bahia Blanca estuary, in order to determine whether its port activities change the beaches’ ecosystems. Although the results are still preliminary, they are encouraging. Addressing the data transmission in UWASN opens new opportunities for studying several environments that have been difficult to address for researchers. Particularly, underwater environments can be easily monitored with sensor networks that use the proposed MAC protocol.

As part of the future work, we intend to evaluate the performance of the proposed protocol in a real-world scenario: throughput, latency and delay against other protocols, like MACA or MACAW. Depending on the obtained results, we will determine specific opportunities and restrictions to use this proposal in physical scenarios.

## Figures and Tables

**Table 1:** Propagation delay.

Distance (m)	Propagation Delay (s)
50	0.0333
100	0.0667
200	0.1333
500	0.3333
1000	0.6666
